# Antibacterial and Mode of Action of Extracts from Endophytic Fungi Derived from *Terminalia mantaly*, *Terminalia catappa*, and *Cananga odorata*

**DOI:** 10.1155/2021/6697973

**Published:** 2021-07-15

**Authors:** Michele Ines Kanko Mbekou, Darline Dize, Victorine Lorette Yimgang, Fred Djague, Rufin Marie Kouipou Toghueo, Norbert Sewald, Bruno Njakou Lenta, Fabrice Fekam Boyom

**Affiliations:** ^1^Department of Biochemistry, Faculty of Science, University of Yaoundé I, PO Box 812, Messa-Yaoundé, Cameroon; ^2^Department of Chemistry, Organic and Bioorganic Chemistry, Bielefeld University, PO Box 100131, D-33501 Bielefeld, Germany; ^3^Department of Chemistry, Higher Teacher Training College, University of Yaoundé 1, PO Box 47, 237 Yaoundé, Cameroon

## Abstract

Emerging drug-resistant bacteria creates an urgent need to search for antibiotics drugs with novel mechanisms of action. Endophytes have established a reputation as a source of structurally novel secondary metabolites with a wide range of biological activities. In the present study, we explore the antibacterial potential of endophytic fungi isolated from different tissues of *Terminalia mantaly*, *Terminalia catappa*, and *Cananga odorata*. The crude ethyl acetate extracts of 56 different endophytic fungi were screened against seven bacterial strains using the broth microdilution method. The antibacterial modes of action of the most active extracts (04) were evaluated using *E. coli* ATCC 25922 and *H. influenzae* ATCC 49247 strains. Both the DPPH and FRAP assays were used to investigate their antioxidant activity, and their cytotoxicity against the Vero cell line was evaluated using the MTT assay. Out of the 56 crude extracts tested, about 13% were considered very active, 66% partially active, and 21% nonactive against all tested bacterial strains with MIC values ranging from 0.32 *μ*g/mL to 25 *μ*g/mL. The four more potent extracts (MIC <5 *μ*g/mL) (from *Aspergillus* sp. N454, *Aspergillus* sp. N13, *Curvularia* sp. N101, and *Aspergillus* sp. N18) significantly lysed the bacteria cells, increased outer membrane permeability, reduced salt tolerance, and inhibited bacterial catalase activity. They exhibited a DPPH free radical scavenging activity with IC_50_ ranging from 150.71 to 936.08 *μ*g/mL. Three of the four potent extracts were noncytotoxic against the Vero cells line (CC_50_ > 100 *μ*g/mL). Results from this investigation demonstrated that endophytes from Cameroonian medicinal plants might content potent antibacterial metabolites. The bioguided fractionation of these potent extracts is ongoing to isolate and characterise potential active ingredients.

## 1. Introduction

Pneumonia, a type of acute respiratory tract infection that affects the lungs, is considered one of the significant public health concerns. This disease is caused by several infectious agents, including bacteria, viruses, fungi, and parasites with bacteria responsible for most cases of mortality and morbidity. In fact, in 2017, pneumonia caused by bacteria was responsible for 808 694 children's death worldwide, with most of the cases reported in developing countries [1, 2]. For instance, pneumonia was responsible for 33.5% of morbidity among children under-five at the Wondo Genet district in Ethiopia [3] and 11.5% of deaths of children under-five in Cameroon in 2018 [4]. For the management of pneumonia, vaccines for the prevention (23-valent pneumococcal polysaccharide vaccine (PPV23), pneumococcal conjugate vaccine (PCV 13)) are available despites their limited protection [5]. On the other hand, antibiotics recommended and usually prescribed for the treatment are costly, inaccessible by the indigenous population, have several undesirables side effects, and more importantly, there is an increasing loss of effectiveness due to the emergency multidrug-resistant bacteria strains [1, 6]. Therefore, it is crucial and urgent to search for novel, more effective, less toxic, and safe antibiotic drugs with multiple modes of action to fight pathogens resistance [7].

Over the past two decades, endophytic fungi have been reported as excellent sources of structurally novel and bioactive secondary metabolites which can constitute perfect starting points for developing new potential antibiotic drugs. Indeed, metabolites produced by endophytic fungi belong to several classes, including alkaloids, lignans, terpenoids, flavonoids, tannins, and steroids, and exhibit multiple biological activities including antibacterial [8–12]. Moreover, our previous study showed that extracts from some endophytic fungi from *Terminalia catappa*, *Terminalia mantaly*, and *Cananga odorata*, three Cameroonian medicinal plants exhibit potent inhibition against various pathogenic bacteria and parasites strains [13]. Therefore, we hypothesized that extracts from these endophytic fungi may produce active metabolites against bacteria pathogens causative agents of human pneumonia. The present study was undertaken to investigate the antibacterial and the modes of action of endophytic fungi extracts isolated from the plants mentioned above.

## 2. Materials and Methods

### 2.1. Endophytic Fungi Isolates and Extracts Preparation

A total of fifty-six isolates (Supplementary material 1) of endophytic fungi from *Terminalia catappa* (51244/HNC), *Terminalia mantaly* (64212/HNC), and *Cananga odorata* (42250/HNC) were used in this study [14]. For extract preparation, each fungal isolate was cultivated by placing agar blocks of actively growing pure culture (3 mm diameter) in a 200 mL Erlenmeyer flask containing 100 mL of Potatoes Dextrose Broth (PDB) medium (Sigma Aldrich, USA). Flask was incubated at 25°C for 7 days. After the incubation period, 100 mL of ethyl acetate was added to each culture, shaken, and kept overnight at room temperature. The mixture was then transferred to a separatory funnel, the organic phase was collected, and the solvent was then removed at 70°C using a rotary vacuum evaporator (Heidolph, Germany). The dry solid residue was prepared at 25 mg/mL in DMSO 100% (Loba Chemie, India) and kept at 4°C before the antibacterial screening. The positive controls Amoxicillin and Ciprofloxacin were prepared at 2 mg/mL in sterile distilled water.

### 2.2. Bacteria and Culture Conditions

The bacteria strains used in this investigation were obtained from the American Type Culture Collection (ATCC) including *Streptococcus pneumoniae* ATCC 49619, *Klebsiella pneumoniae* ATCC 13883, *Staphylococcus aureus* BAA-977, *Staphylococcus aureus* ATCC 43300, *Haemophilus influenzae* ATCC 49247, *Escherichia coli* ATCC 25922, and *Pseudomonas aeruginosa* HM 601. Twenty-four hours before each experiment, bacteria were subcultured on nutrient agar tubes at 37°C.

### 2.3. Determination of Minimum Inhibitory Concentration (MIC) of Extracts

The minimum inhibitory concentration (MIC) of extracts was determined according to the M07-A9 Clinical Laboratory Standards Institute microdilution method using 96-wells microtitre plates [15]. Briefly, 4 *μ*L of extracts and reference drugs (Amoxicillin and Ciprofloxacin) from stock solutions were introduced in the well followed by the addition of 96 *μ*L of bacteria inoculum standardised at 10^6^ CFU/mL. A blank column was included for sterility control, while bacterial strains in the culture medium without any inhibiting substance were negative control. The concentrations of extracts ranged from 0.195 *μ*g/mL to 25 *μ*g/mL, and that of Ciprofloxacin and amoxicillin ranged from 0.562 *μ*g/mL to 128 *μ*g/mL. After 24 hours of incubation at 37°C, the turbidity was observed as an indication of growth. MIC was defined as the lowest concentration inhibiting the visible growth of bacteria. All tests were performed in triplicate.

### 2.4. Determination of Possible Modes of Action of Promising Extracts

#### 2.4.1. Measurement of the Lytic Activity of Extracts

The determination of extracts' lytic activity was performed as described by Limsuwan and Voravuthikunchai [16]. An overnight bacterial culture was used to prepare bacterial suspension at 0.5 Mc Farland in NaCl 0.9%. The bacterial suspension was treated with extracts at MIC, 2 MIC, and 4 MIC and incubated at 37°C. The optical density (OD) at 620 nm was measured at four different periods, including 0 h, 1 h, 2 h, and 4 h using the microplate reader Infinite M200 (TECAN). A decrease in OD 620 nm indicated bacterial cell lysis. Corresponding dilutions of the extract were used as blanks. Ciprofloxacin at 2 *μ*g/mL was used as a positive control. The results were expressed as a ratio of the OD at each time interval versus the OD at 0 min (in %). All assays were carried out in triplicate.

#### 2.4.2. Integrity of the Cell Membrane

The integrity of the cell membrane of *Haemophilus influenzae* ATCC 49247 and *Escherichia coli* ATCC 25922 was carried out as previously described by Carson et al. [17] with slight modifications. Briefly, the test bacteria in the exponential growth phase were washed and suspended in sterile peptone water (0.1 g/100 mL). The bacterial strains (5.10^7^ CFU/mL) were incubated with extracts at 4 MIC for different periods (0, 30, 60, 90, and 120 min). The mixtures were then centrifuged at 5000 rpm for 10 minutes, after which the UV absorbance of the supernatant was measured at 260 nm using the microplate reader Infinite M200 (TECAN). The untreated bacterial cultures in sterile peptone water served as the negative control. Ciprofloxacin at 2 *μ*g/mL was a positive control, and each test was performed in triplicate. Results were expressed in terms of the optical density of 260 nm absorbing materials in each interval for the ultimate time.

#### 2.4.3. Outer Membrane Permeability Assay

Outer membrane (OM) permeability of *H. influenzae* ATCC 49247 and *E. coli* ATCC 25922 was determined according to the method described by Oliviera et al. [18] with small modifications. An overnight culture (5.10^7^ CFU/mL) was inoculated into nutrient broth containing the extracts at MIC, 2MIC, and 4 MIC. The media was then poured into sterilised 96-well microplates (100 *μ*L) and incubated at 37°C for 24 h. After the incubation time, the growth of *H. influenzae* ATCC 49247 and *E. coli* ATCC 25922 was measured at 450 nm using the Microplate Reader Infinite M200 (TECAN). The graph of bacterial growth parameter (OD/450 nm) in the function of extract concentration (*μ*g/mL) was plotted. Ciprofloxacin (concentration ranged from 1 to 4 *μ*g/mL) was used as positive control, and each test was conducted in triplicate.

#### 2.4.4. Salt Tolerance Assay

The salt tolerance effect of *H. influenzae* ATCC 49247 and *E. coli* ATCC 25922 treated with the MIC concentration of the extracts was evaluated on nutrient agar plates supplemented with different potassium concentrations chloride (KCl) [19]. 24 h before the experiment, bacteria strains were cultured on a nutrient agar medium and incubated at 37°C. The overnight culture was treated with extracts at 4 MIC and further incubated for 60 min at 37°C. The samples were then serially diluted (Dilution Factor = 100) and inoculated on nutrient agar plates supplemented with different concentrations of KCl (0%, 2.5%, 5.0%, and 10.0%). Each plate was incubated for 24 hours at 37°C. Ciprofloxacin at 4 *μ*g/mL was used as a positive control. After the incubation period, both the controls and treated plates were compared, the colonies were counted, and the results were expressed in terms of Log10 CFU/mL. The experiment was performed in triplicate.

#### 2.4.5. Inhibition of Catalase Activity

The catalase inhibitory activity of the extracts was evaluated using the protocol described by Weydert and Cullen [20] with slight modifications. Extracts at the MIC concentration were added in a test tube containing 400 *μ*L of hydrogen peroxide (40 mM) and 400 *μ*L of PBS. The mixture then transferred in another tube containing 200 *μ*L of a bacterial suspension (1.5 × 10^8^ UFC/mL). The samples were incubated at 37°C for 30 min; after which, they were centrifuged at 1200 rpm for 10 min. The supernatants were collected, and their optical density (OD) read at 232 nm. The phosphate buffer constituted the blank, while bacterial strains in phosphate buffer without any inhibiting substance was used as a negative control. The mixture of Ciprofloxacin, phosphate buffer, and bacterial strain constituted positive control. The percentage of remaining hydrogen peroxide was determined according to the following formula (1).

(1) $$\begin{array}{c} \%\text{of remaining H}2\text{O}2=\frac{\left(A_{\text{sample}}-A_{\text{negative control}}\right)\times 100}{A_{\text{negative control}}\;}. \end{array}$$

*A*_negative control_ is the absorbance of H_2_O_2_ without the extract, and *A*_sample_ is the absorbance of H_2_O_2_ with the extract.

### 2.5. Evaluation of the *In Vitro* Antioxidant Potential and Cytotoxicity of Promising Extracts

#### 2.5.1. DPPH Radical Reduction Assay

DPPH (1, 1-diphenyl-2-picrylhydrazyl) radical scavenging assay was performed according to the method described by Scherer and Godoy [21]. Briefly, 25 *μ*L of extracts dissolved in methanol were added to wells of a microtiter plate in triplicate followed by 75 *μ*L of DPPH solution (0.01%) to yield extract solution concentration ranging from 1000 to 1.95325 *μ*g/mL. The content was mixed and incubated for 30 min in the dark at 25 ± 2°C after which the absorbance was taken at 517 nm using a Microplate Reader Infinite M200 (TECAN). Ascorbic acid was used as a standard antioxidant with final concentrations ranging from 25 to 0.195 *μ*g/mL. The results were expressed through the calculation of the DPPH• inhibition percentage according to the following formula (2).

(2) $$\begin{array}{c} \text{Inhibition of DPPH }\left(\%\right)=\frac{\left(A_{\text{control}}-A_{\text{sample}}\right)\times 100}{A_{\text{control}}}, \end{array}$$

where *A*_control_ is the DPPH• radical absorbance without the extract and *A*_sample_ is the DPPH• absorbance with the extract.

The concentration of extract proportional to a 50% inhibition of DPPH• radical (IC_50_) was obtained by analysing the extract solution concentration versus inhibition percentage graphic. Thus, lower extract concentrations (*μ*g/mL) mean greater antioxidant capacity provided by the analysed extract.

#### 2.5.2. Ferric Ion Reducing Antioxidant Power (FRAP) Assay

The ferric reducing antioxidant power of potent extracts was determined using the method described by Benzie et al. [22]. 25 *μ*L of extracts at different concentrations (7.8125-4000 *μ*g/mL) were introduced into a microtiter plate, and 25 *μ*L of a solution of Fe^3+^ at 1.2 mg/mL were added. The plates were preincubated for 15 min at room temperature, and 50 *μ*L of 0.2% ortho-phenanthrolin were then added to obtain a final extract concentration ranging from 1000 to 1.95325 *μ*g/mL. The reaction mixtures were further incubated for 15 min at room temperature, and the absorbance was measured at 505 nm using a microplate reader Infinite M200 (TECAN) against the blank (made of 25 *μ*L methanol+25 *μ*L Fe3++50 *μ*L ortho-phenanthrolin). Ascorbic acid was used as positive control and tested at concentrations ranging from 0.103 to 6.60 *μ*g/mL. The assay was performed in triplicate.

#### 2.5.3. Evaluation of the Cytotoxicity of Promising Extracts

The cytotoxic effect of antibacterial extracts was assessed using the MTT assay [23], targeting normal monkey kidney Vero cells ATCC CRL1586 cultured in complete medium containing 13.5 g/L DMEM (Gibco, Waltham, MA USA), 10% fetal bovine serum (Gibco, Waltham, MA, USA), 0.21% sodium bicarbonate (Sigma-Aldrich, New Delhi, India), and 50 *μ*g/mL gentamicin (Gibco, Waltham, MA, USA). Essentially, Vero cells at 5 × 10^3^ cells/200 *μ*L/well were seeded into 96-well flat-bottomed tissue culture plates (Corning, USA) in complete medium. Fifty microliters of serially diluted extract solutions (≤200 *μ*g/mL) were added after 24 h of seeding and cells plus test substance incubated for 48 h in a humidified atmosphere at 37°C and 5% CO_2_. DMSO (0.4% (*v*/*v*) was added as negative control (100% growth). Twenty microliters of a stock solution of MTT (5 mg/mL in 1x phosphate-buffered saline) were added to each well, gently mixed, and incubated for an additional 4 h. After spinning the plate at 1500 rpm for 5 min, the supernatant was carefully removed and 100 *μ*L of 100% DMSO (*v*/*v*) was added to dissolve the formazan. The plate was read on a microtiter plate reader (Infinite M200 (TECAN)) at 570 nm. The 50% cytotoxic concentrations (CC_50_) of extracts were determined by analysis of the dose-response curves.

### 2.6. Statistical Analysis

Data collected from at least three independent experiments were analysed using One-Way ANOVA using Graph Pad Prism 5. Data are expressed as mean ± SD of experiments performed in triplicate. Error bars represent the SD, and a, b, and c represent *p* < 0.05, ^∗∗^*p* < 0.001, ^∗∗∗^*p* < 0.0001, respectively, significant difference compared to untreated sample.

## 3. Results

### 3.1. Extraction Yield and Antibacterial Potency of Endophytic Extracts

**Figure 1:**
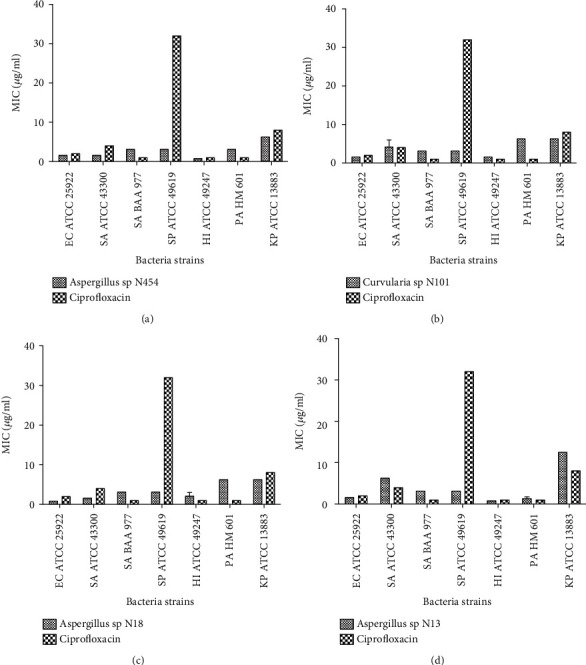
Minimal inhibitory concentration (MIC) of the most potent extracts on bacteria strains. The errors bars represent the standard deviation of measurement of a sample in three separate runs. (a) *Aspergillus* sp. N454. (b) *Curvularia* sp. N101. (c) *Aspergillus* sp. N18. (d) *Aspergillus* sp. N13. SA: *Staphylococcus aureus*; EC: *Escherichia coli*; SP: *Streptococcus pneumoniae*; HI: *Haemophilus influenzae*; PA: *Pseudomonas aeruginosa*; KP: *Klebsiella pneumoniae*.

The extraction yield of endophytic fungi ranged from 52 to 200 mg for 200 mL of culture medium, with the *unidentified fungal* N445 from *C. odorata* root producing the higher amount of metabolites (Supplementary material 2). Each of the fifty-six extracts was screened for antibacterial activity against seven pathogenic bacteria. The MIC ranged from 0.39 to >25 *μ*g/mL depending on the fungal extract and the tested pathogen (Supplementary material 2). Our criteria for the activity of extracts against bacteria were defined as follows: very active (MIC <5 *μ*g/mL), partially active (MIC 5-15 *μ*g/mL), and nonactive (MIC >15 *μ*g/mL). Therefore, out of the 56 extracts, eight were very active (13%) with the more potent (MIC <5 *μ*g/mL) being extracts from *Aspergillus* sp. N454, *Aspergillus* sp. N18, *Curvularia* sp. N101, and *Aspergillus* sp. N13 isolated, respectively, from *C. odorata* and *T. catappa* (Figure 1). Thirty-seven extracts (66%) showed moderate potency with MIC between 5 and 15 *μ*g/mL, while 12 extracts (21%) with MIC >15 *μ*g/mL were considered as inactive. Overall, endophytic *Aspergillus* spp. exhibited the best activity (MIC ranged from 0.78 to 6.25 *μ*g/mL), while *E. coli* ATCC 25922 and *H. influenzae* ATCC 49247 were the more sensitive pathogenic strains. To continue our investigation, the four most prominent crude extracts, including *Aspergillus* sp. N454, *Aspergillus* sp. N18, *Curvularia* sp. N101, and *Aspergillus* sp. N13 (Figure 1), were selected for the mode of action and antioxidant studies.

### 3.2. The Mode of Action of Promising Extracts

#### 3.2.1. Bacteriolytic Effect of Selected Extracts

**Figure 2:**
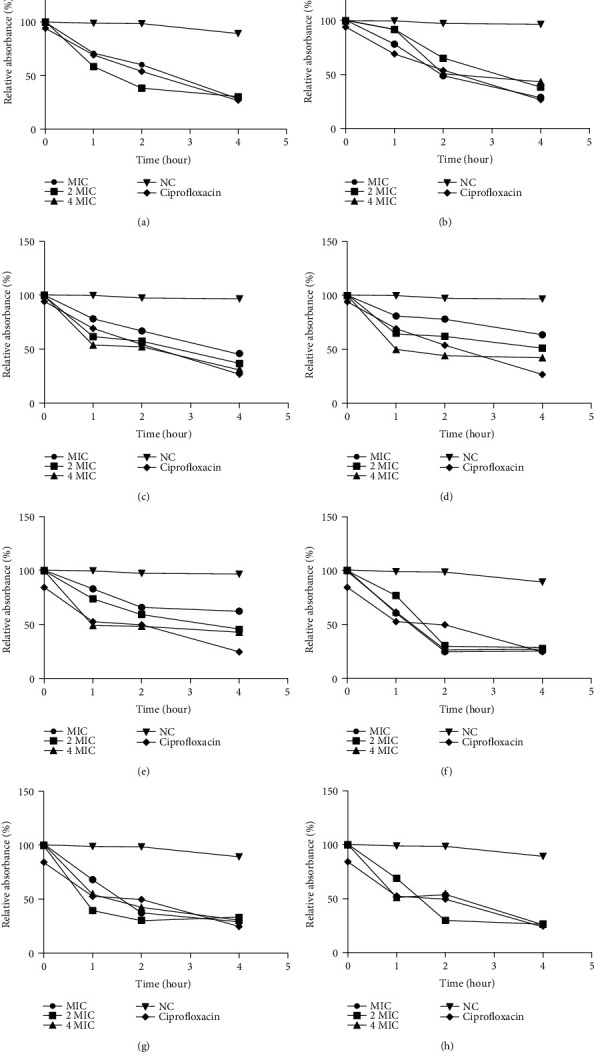
Bacteriolytic activity of endophytic fungi extracts on *E. coli* ATCC 25922 and *H. influenzae* ATCC 49247. (a) *Aspergillus* sp. N454 on *E. coli*. (b) *Aspergillus* sp. N18 on *E. coli*. (c) *Aspergillus* sp. N13 on *E. coli*. (d) *Curvularia* sp. N101 on *E. coli*. (e) *Aspergillus* sp. N454 on *H. influenzae*. (f) *Aspergillus* sp. N18 on *H. influenzae*. (g) *Aspergillus* sp. N13 on *H. influenzae*. (h) *Curvularia* sp. N101 on *H. influenzae*. Data are expressed as the mean ± SD. MIC: minimal inhibitory concentration; NC: negative control.

The bacteriolysis assay was performed to investigate if active extracts inhibit *H. influenzae* ATCC 49247 and *E. coli* ATCC 25922 through cell lysis. A viable bacteria absorb light at 620 nm; therefore, bacteriolysis occurs if the optical density of the medium at 620 nm decreased with time [17]. The cell lysis activity exhibited by fungi extracts is summarised in Figure 2. Globally, the bacterial cells' treatment with fungal extracts caused gram-negative bacteria cell lysis at all tested concentrations. This cell lysis was more significant than 50% and 55% for *E. coli* and *H. influenzae*, respectively, after 4 hours of incubation at 2 MIC and 4 MIC depending on the tested extract. The reduction of the bacterial population was more drastic in the case of *H. influenzae* than *E. coli*. Overall, the bacteriolysis was higher with an extract from *Aspergillus* sp. N18 (71%) on *E. coli* at 4 MIC and *Curvularia* sp. N101 (75%) on *H. influenzae* at 2 MIC, respectively.

#### 3.2.2. The Effect of Extracts on the Integrity of the Cell Membrane

**Figure 3:**
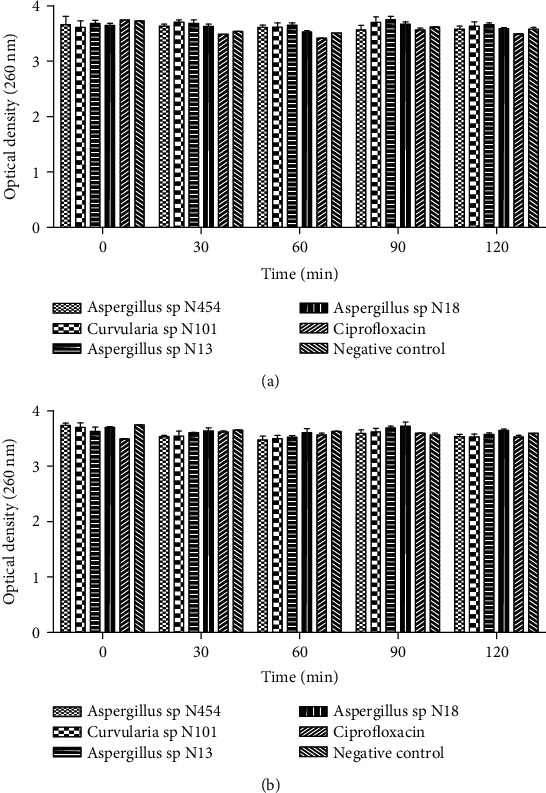
Total nucleotide leakage from (a) *Escherichia coli* ATCC 25922 and (b) *Haemophilus influenzae* ATCC 49247 treated with different endophytic fungi extracts at their MIC concentration. Data are expressed as the mean ± SD.

The cytoplasmic membrane's integrity was analysed by determining the release of cellular materials including nucleic acids, proteins, metabolites, and ions, which were absorbed at 260 nm into the bacterial suspensions. Treatment of *H. influenzae* ATCC 49247 and *E. coli* ATCC 25922 with potent fungal extracts at the MIC concentrations indicated no significant cell leakage of 260 nm absorbing material in a time-dependent manner. This absence of nucleotide leakage was observed until the 120^th^ minute of incubation (Figures 3(a) and 3(b)). The same result was obtained in the control group treated with Ciprofloxacin, revealing its disability to damage the tested bacteria strains' cytoplasmic membrane.

#### 3.2.3. The Effect of Extracts on the Permeability of Outer Cell Membrane

**Figure 4:**
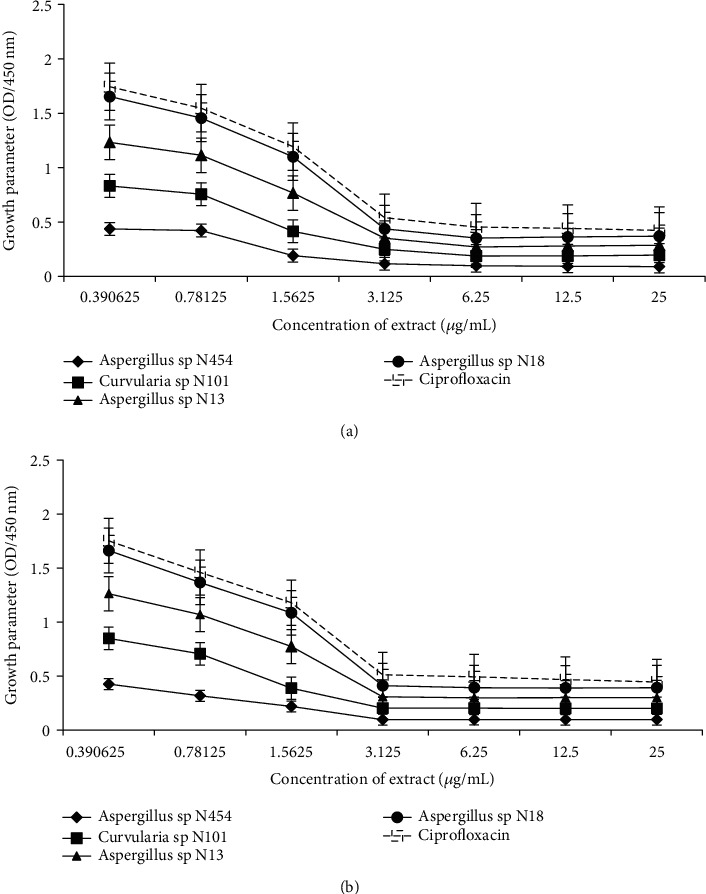
Effect of endophytic fungi extracts on the membrane permeability of (a) *H. influenzae* ATCC 49247 and (b) *E. coli* ATCC 25922. Data are expressed as the mean ± SD.

Bacterial cell membrane permeability was determined in terms of optical density at 450 nm (Figures 4(a) and 4(b)). Measurement of the optical density of bacterial cells treated with endophytes extracts demonstrated that extracts from *Aspergillus* sp. N454, *Aspergillus* sp. N18, *Aspergillus* sp. N13, and *Curvularia* sp. N101 affect the permeability of *E. coli* ATCC 25922 and *H*. *Influenzae* ATCC 49247. All extracts resulted in a decrease of the optical density at 450 nm, which indicated leakage of intracellular components, including electrolytes from the cells. Furthermore, in both treated bacteria, when the concentration of extracts increases from 0.39 to 3.125 *μ*g/mL, there was a sharp decrease in the optical density which corresponds to the loss of viability of tested bacteria (Figures 4(a) and 4(b)). The inhibition of the growth of *H. influenzae* and *E. coli* was more intense when treated with an extract from *Aspergillus* sp. N454 as compared to antibiotic Ciprofloxacin at almost all the concentrations tested.

#### 3.2.4. The Effect of Extracts on the Loss of Salt Tolerance Capacity

**Figure 5:**
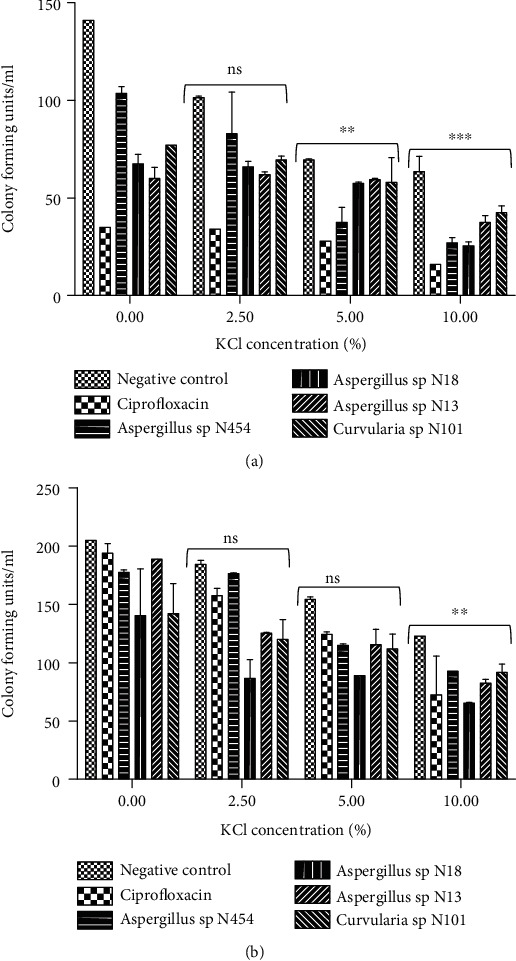
Effect of potent extracts on reducing salt tolerance of (a) *E. coli* ATCC 25922 and (b) *H. influenzae* ATCC 49247 at the MIC concentration. The error bars represent the standard deviation of measurement of a sample in three separate sample runs. ns: nonsignificant; ^∗∗∗^: significantly different compared to the untreated samples (*p* < 0.001); ^∗∗^: significantly different compared to the untreated samples (*p* < 0.01).

The effect extracts on salt tolerance of bacteria are summarised in Figures 5(a) and 5(b). The addition of KCl to nutrient agar (NA) medium significantly reduced the colony-forming units of treated *E. coli* ATCC 25922 and *H. influenzae* ATCC 49247. *H. influenzae* treated with KCl and fungal extract revealed that the number of bacteria able to form colonies on NA-KCl was not significantly reduced at 2.5% and 5% of KCl concentration compared to the control (0% KCl), while, at 10% of KCl, a minimal bacterial growth was recorded. However, regarding *E. coli*, the proportion of bacteria able to form colonies on KCl nutrient agar was significantly reduced when KCl was used at 5% and 10% g/L. In general, the reduction of colonies formation while treated with extracts was proportional to the medium's KCl concentration.

#### 3.2.5. The Effect of Extracts on the Catalase Activity

**Figure 6:**
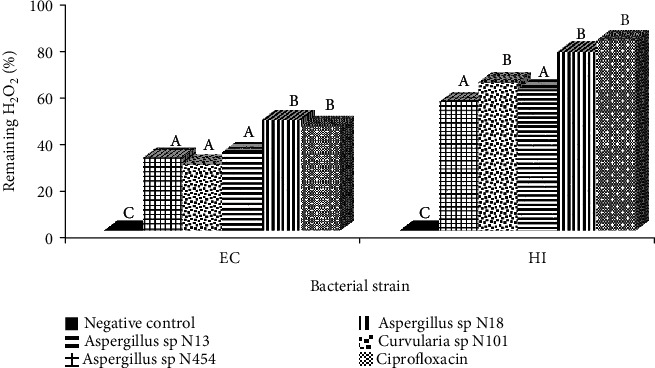
Effect of endophytic fungi extracts on catalase activity of *Escherichia coli* and *Haemophilus influenzae* at the MIC concentration. Data are expressed as the mean ± SD. Values with different letters express a significant difference at *p* < 0.05. EC: *Escherichia coli*; HI: *Haemophilus influenzae.*

The inhibition of the catalase activity of *E. coli* and *H. influenzae* was evaluated by comparing the amount of H_2_O_2_ remaining in the medium after the addition of fungal extracts to the control. The results obtained were summarised in Figure 6 below. The percentages of remaining H_2_O_2_ in bacteria culture treated with extracts were ranged from 28.48 to 47.63% for *E. coli* and from 55.28 to 76.67% for *H. influenzae* highlighting the ability of tested extracts to exert a certain degree of inhibition against the activity of the bacterial catalase enzyme. The crude extract from *Aspergillus* sp. N18 exerted the highest degree of inhibition against *H. influenzae* (%of remaining H_2_O_2_ = 47.63) and *E. coli* (%of remaining H_2_O_2_ = 76.67). The catalase inhibition activity exhibited by *Aspergillus* sp. N18 is comparable to that of the positive control, Ciprofloxacin showing a percentage of remaining H_2_O_2_ of 45.09 and 81.57% on *H. influenzae* and *E. coli*, respectively.

### 3.3. Antioxidant Potential and Cytotoxicity of Selected Extracts

To understand the effect of active extracts to fight against oxidative stress, the DPPH and the Fe^3+^ reducing ability of extracts were evaluated (Table 1). The results show that the inhibition concentration 50 (IC_50_) of DPPH ranged from 150.71 to 936.08 *μ*g/mL depending on tested extracts. Extract of *Aspergillus* sp. N454 showed a high scavenging activity with IC_50_ of 150.71 *μ*g/mL, whereas the extract from *Curvularia* sp. N101 showed the least antioxidant activity (IC_50_ > 1000 *μ*g/mL). Concerning the Fe^3+^-reducing power assay, only the ethyl acetate extract from *Aspergillus* sp. N13 exhibited activity, although very weak with RC_50_ of 760.96 *μ*g/mL (Table 1).

The cytotoxicity of the extracts was tested against Vero cells ATCC CRL1586. As shown in Table 1, extract from *Aspergillus* sp. N13 was cytotoxic against Vero cells ATCC CRL1586 (CC_50_ of 14.285 *μ*g/mL), while no cytotoxic activity was observed with other tested extracts (CC_50_ > 100 *μ*g/mL). These values were within the cutoff point of the National Cancer Institute criteria for noncytotoxicity crude extract (IC_50_ < 20 *μ*g/mL).

## 4. Discussion

The antibacterial activity of the crude ethyl acetate extracts of different endophytic fungi isolated from *Cananga odorata*, *Terminalia mantaly*, and *Terminalia catappa* was evaluated with the aims of identifying potential active extract(s) that can be further investigated for the discovery of active principle useful in the treatment of lower respiratory tract infection as pneumonia. Endophytic fungi have enormous potential to produce an extensive range of bioactive secondary metabolites with therapeutic activity such as antibacterial, antifungal, and antioxidant [10–12]. In the present study, a total of 8 (13%) strains showed excellent antibacterial activity with a broad spectrum among which extracts from endophytic fungi *Aspergillus* sp. N454, *Aspergillus* sp. N13, *Aspergillus* sp. N18, and *Curvularia* sp. N101 was more active against all tested bacteria. Literature reveals that the fungi from *Aspergillus* and *Curvularia* genera can produce antimicrobial compounds [24, 25]. In the present work, the antibacterial mode was done to understand the mechanism of inhibition of active extracts against bacteria (*H. influenzae* and *E. coli*). The bacteriolysis assay results showed that the crude extracts' inhibitory effect could be related to bacterial cell lysis. Positive results obtained with endophytic fungi extracts suggested that each extract's active metabolites content might have entered into the bacterial cell through bacterial porins. They might have affected the bacterial enzymes, causing cell lysis and death [26]. Additionally, the cell lysis observed may also have been due to the weakening of the cell wall and the subsequent rupture of the cytoplasmic membrane due to osmotic pressure rather than a specific action on the cytoplasmic membrane [17].

Further, the action of extracts on the permeability of the bacterial outer membrane was studied. The permeability of the outer membrane of *E. coli* ATCC 25922 and *H. influenzae* ATCC 49247 was significantly impaired by the addition of fungal extracts into the culture as indicated by the decrease of the relative absorbance in the bacterial solution. Gram-negative bacteria's outer membrane is made by lipoproteins, phospholipids, and vital lipopolysaccharide molecules, which confer to her impermeability to various antibacterial agents [27]. The control of the cellular membrane's permeability is a crucial regulatory factor for various cellular functions, including cell metabolism maintenance, solute transport, and energy transduction processes [28, 29]. These potent extracts may content hydrophobic compounds which enhanced their accumulation inside periplasmic space inducing the degradation of some necessary bacterial enzymes such as alkaline phosphatase leading to cellular lysis [17, 30–32].

Additionally, extracts may have altered the bacterial cell's permeability by reducing its osmoregulatory ability to penetrate or exclude toxic materials, resulting in their loss of salt tolerance [19]. This hypothesis is supported by the fact that the treatment of *E. coli* and *H. influenzae* with fungal extracts reduced the bacteria's ability to form colonies on media containing KCl. The studies on salt tolerance of bacteria were also reported by many investigators [17, 33, 34]. The leakage of the 260 nm absorbing material was evaluated to see whether potent extracts were also acting on the bacterial inner membrane. Unfortunately, *E. coli* ATCC 25922 and *H. Influenzae* ATCC 49247 treated with endophytic fungi extracts did not significantly lose 260-nm-absorbing materials, suggesting nucleic acids were not lost through a damaged cytoplasmic membrane. The inactivity of extracts on the inner membrane compare to the outer membrane can be explained by their difference in composition which is essentially made of phospholipids. Previous reports demonstrated that antimicrobial compounds such as chlorhexidine, hexachlorophene, phenethyl alcohol, tetracyclines, and polymyxin that act on cytoplasmic membrane induced structural and functional abnormalities of the bacterial membrane phospholipids bilayer [17, 35].

Inhibition of catalase activity of an extract can indicate the reduction of pathogen resistance towards oxidative stress. Several pathogens produced catalase in order to defend themselves against attacks by hydrogen peroxide, a weapon commonly used by macrophages, in addition to oxidative stress [36]. Fungal extracts tested revealed a high inhibition of catalase activity of *E. coli* and *H. influenzae* indicating that those extracts can be used to prevent DNA damage caused by hydroxyl radicals (OH-) issued from the decomposition of hydrogen peroxide by pathogenic bacteria [36].

The potential of extracts to prevent oxidative stress in human was also investigated through the DPPH radical scavenging assay and FRAP reducing power. Antioxidants are known to prevent oxidative stress-mediated toxicity caused by oxygen-free radicals [37]. It was evident from the results that the fungal extracts contain radical scavenging compounds as previously reported [13, 38, 39]. Iron is essential for life because it is required for many enzymes' activity, oxygen transport, and respiration. However, iron is an extremely reactive metal and catalyses oxidative changes in lipids, proteins, and other cellular components. It causes lipid peroxidation through the reaction and decomposes the lipid hydroxide into peroxyl and alkoxyl radicals that can perpetuate the chain reactions [40]. From this study, it may be noted that only endophytic fungal extract from *Aspergillus* sp. N13 showed promising reducing potential indicating its antioxidant potential.

Results obtained are precise concerning the noncytotoxicity of fungal extracts from *Aspergillus* sp. N454, *Aspergillus* sp. N18, and *Curvularia* sp. N101. However, *Aspergillus* sp. N13 exhibited high cytotoxic activity against Vero cell. This cytotoxicity could be attributed to the quality and quantity of compounds produced by this isolate. Endophytic fungi, particularly from the *Aspergillus* genus, have been reported as excellent producers of strong cytotoxic metabolites [41]. The difference observed in cytotoxicity found among active extracts from the *Aspergillus* genus could be due to culture conditions that could have favored or unfavored toxic compounds' production [42, 43]. For instance, our previous investigation of endophytic fungal *Aspergillus* sp. 58 isolated from the bark of *T. catappa* showed that ethyl acetate extract obtained from the culture in PDB was nontoxic; however, when the growth medium was supplemented with DMSO, extract obtained was highly cytotoxic against HEK293T cell line [44]. Watanabe et al. [45] also reported that *A. fumigatus* cultured produced toxic gliotoxins compounds in the presence of high oxygen concentration. Kamei et al. [46] also reported the production of cytotoxic molecules produced by *A. fumigatus* when cultured in the presence of macrophages.

## 5. Conclusion

In this study, ethyl acetate extracts of endophytic fungi derived from *T. catappa*, *T. mantaly*, and *C. odorata* demonstrated strong antibacterial activity against pathogenic bacteria implicated in pneumonia. Results indicated that fungal isolates of *Aspergillus* sp. N454, *Aspergillus* sp. N18, and *Curvularia* sp. N101 may be useful as an alternative to producing antibacterial drugs. Therefore, further investigation to isolate and identify the bioactive compounds responsible for these fungi's specified biological activities is currently ongoing.

## Figures and Tables

**Table 1:** DPPH radical (IC_50_) and RC_50_ of fungi extract and cytotoxicity (CC_50_). Means ± SD.

Fungi name	IC_50_ (*μ*g/mL)	RC_50_ (*μ*g/mL)	CC_50_ (*μ*g/mL)
*Aspergillus* sp. N13	250 ± 0.350^c^	760 ± 0.07^b^	14.28 ± 5.86
*Curvularia* sp. N101	>1000	>1000	>100
*Aspergillus* sp. N454	150.71 ± 0.02^b^	>1000	>100
*Aspergillu*s sp. N18	936.08 ± 1.93^d^	>1000	>100
Ascorbic acid	2.71 ± 0.08^a^	13.94 ± 0.27^a^	—

Values carrying the same letter superscripts are not significantly different (*p* > 0.05). IC_50_: inhibitory concentration 50 of DPPH radical; RC_50_: Fe^3+^-reducing concentration 50; CC_50_: cytotoxic concentration 50; SD: standard deviation. Data are presented as mean values ± standard deviation of triplicate experiments.

## Data Availability

Data used to support the findings of this study are all included within the article.

## Supplementary Materials

Supplementary Materials Supplementary material 1: endophytic fungi: their host plant, isolation site and names. Supplementary material 2: extraction yield (mg/200 mL), and mimimal inhibitory concentration (*μ*g/mL (mean ± SD)) of ethyl acetate extracts from the 56 endophytic fungi used in the present investigation.
