# Supplementary material for: Antibacterial and Mode of Action of Extracts from Endophytic Fungi Derived from Terminalia mantaly, Terminalia catappa, and Cananga odorata
Source: Biomed Res Int. 2021 Jul 15;2021:6697973. doi: 10.1155/2021/6697973 (PMC8302376; doi:10.1155/2021/6697973)
Supplement: Supplementary Materials — Supplementary material 1: endophytic fungi: their host plant, isolation site and names. Supplementary material 2: extraction yield (mg/200 mL), and mimimal inhibitory concentration (μg/mL (mean ± SD)) of ethyl acetate extracts from the 56 endophytic fungi used in the present investigation. [file 6697973.f1.pdf]

Antibacterial and mode of action of extracts from endophytic fungi derived from *Terminalia mantaly*, *Terminalia catappa* and *Cananga odorata*

Michele Ines Kanko Mbekou[1], Darline Dize[1], Victorine Lorette Yimgang[1], Fred Djague[1] , Rufin Marie Kouipou Toghueo[1], Norbert Sewald[2], Bruno Njakou Lenta[3], Fabrice Fekam Boyom[1*]

[1]Department of Biochemistry, Faculty of Science, University of Yaoundé I, P.O. Box 812, Messa-Yaoundé, Cameroon

[2]Department of Chemistry, Organic and Bioorganic Chemistry, Bielefeld University, P.O. Box 100131, D-33501 Bielefeld, Germany

[3]Department of Chemistry, Higher Teacher Training College, University of Yaoundé 1, P.O. Box 47, 237 Yaoundé, Cameroon

*Correspondence: fabrice.boyom@fulbrightmail.org

Supplementary material 1: Endophytic fungi: their host plant, isolation site and names

**Plant name**	**Site of isolation**	**Fungi isolate**	**Fungal name**
Cananga odorata	Petals	N431	*Unidentified fungal*
		N422	*Unidentified fungal*
		N399	*Unidentified fungal*
		N419	*Unidentified fungal*
		N401	*Unidentified fungal*
	Flowers	N478	*Unidentified fungal*
	Ribs	N454	*Aspergillus* sp
	Leaves	N330	*Diaporthe phaseolorum*

	Stem	N284	*Unidentified fungal*
		N283	*Unidentified fungal*
		N298	*Diaporthaceae* sp
		N289	*Unidentified fungal*
	Bark	N241	*Fungal* sp.
		N262	*Unidentified fungal*
		N263	*Unidentified fungal*
		N266	*Unidentified fungal*
		N251	*Unidentified fungal*
		N276	*Unidentified fungal*
		N256	*Unidentified fungal*
		N2541	*Unidentified fungal*
		N240	*Fusarium* sp
		N268	*Fungal* sp
	Root	N441	*Unidentified fungal*
		N448	*Unidentified fungal*
		N445	*Unidentified fungal*
	Root bark	N368	*Unidentified fungal*
	Ribs	N32	*Corynespora* sp

Terminalia catappa	Leaves	N89	*Fungal endophyte* sp
		N114	*Phomopsis* sp
		N108	*Guignardia* sp
	Stem	N75	*Pestalotiopsis* sp
		N74	*Xylaria* sp
		N44	*Unidentified fungal*
		N781	*Paraconiothyrium variabile*
		N51	*Pestalotiopsis* sp
		N58	*Unidentified fungal*
		N44	*Unidentified fungal*
	Bark	N101	*Curvularia* sp
		N97	*Trichoderma* sp
		N81	*Phomopsis* sp
	Root bark	N18	*Aspergillus* sp
		N13	*Aspergillus* sp
		N23	*Unidentified fungal*
		N15	*Unidentified fungal*
		N201	*Colletotrichum* sp

Terminalia mantaly	Ribs	N200	*Fungal* sp
		N191	*Unidentified fungal*
		N196	*Unidentified fungal*
		N221	*Unidentified fungal*
		$N230_2$	*Unidentified fungal*
		$N229_1$	*Diaporthales* sp
	Leaves	N127	*Diaporthales* sp
		N116	*Phoma* sp
		N120	*Xylaria laevis*
	Stem	N162	*Diaporthe* sp
	Bark	$N190_1$	*Unidentified fungal*
		N178	*Unidentified fungal*

14

Supplementary material 2: Extraction yield (mg/200mL), and MIC (µg/mL) of ethyl acetate extracts from endophytic fungi (Mean ±SD)

**Plant name**	**Fungal name**	**Yield**	**EC ATCC 25922**	**SA ATCC 43300**	**SA BAA-977**	**SP ATCC 49619**	**HI ATCC 49247**	**PA HM 601**	**KP ATCC 13883**
Cananga odorata	*Unidentified fungal* N431	104	3.125 ± 0.00	12.5 ± 0.00	3.125 ± 0.00	6.25 ± 0.00	0.78 ± 0.00	3.125 ± 0.00	3.125 ± 0.00
	Unidentified fungal N422	100	2.343 ± 1.11	23.14 ± 0.00	5.787 ± 0.00	3.125 ± 0.00	25 ± 0.00	3.125 ± 0.00	25 ± 0.00
	Unidentified fungal N399	128	0.39 ± 0.00	12.5 ± 0.00	3.125 ± 0.00	3.125 ± 0.00	2.343 ± 1.11	12.5 ± 0.00	12.5 ± 0.00
	Unidentified fungal N419	132	0.78 ± 0.00	5.787 ± 0.00	5.787 ± 0.00	3.125 ± 0.00	>25	1.56 ± 0.00	25 ± 0.00
	Unidentified fungal N401	100	1.17 ± 0.55	6.25 ± 0.00	6.25 ± 0.00	3.125 ± 0.00	1.56 ± 0.00	25 ± 0.00	25± 0.00
	Unidentified fungal N478	120	12.5 ± 0.00	25 ± 0.00	25 ± 0.00	3.125 ± 0.00	25 ± 0.00	12.5 ± 0.00	25 ± 0.00
	Aspergillus sp N454	100	1.56 ± 0.00	1.56 ± 0.90	3.125 ± 0.00	3.125 ± 0.00	0.78 ± 0.00	3.125 ± 0.00	6.25 ± 0.00
	Diaporthe phaseolorum N330	80	1.56 ± 0.00	5.787 ± 0.00	5.787 ± 0.00	3.125 ± 0.00	25 ± 0.00	1.56 ± 0.00	12.5 ± 0.00
	Unidentified fungal N284	106	3.125 ± 0.00	6.25 ± 0.00	6.25 ± 0.00	3.125 ± 0.00	3.125 ± 0.00	6.25 ± 0.00	6.25 ± 0.00
	Unidentified fungal N283	69	0.78 ± 0.00	2.343 ± 1.11	3.125 ± 0.00	3.125 ± 0.00	1.56 ± 0.00	12.5 ± 0.00	12.5 ± 0.00
	Diaporthaceae sp N298	130	1.56 ± 0.00	5.787 ± 0.00	5.787 ± 0.00	3.125 ± 0.00	12.5 ± 0.00	1.56 ± 0.00	12.5 ± 0.00

	Unidentified fungal N289	111	0.78 ± 0.00	12.5 ± 0.00	3.125 ± 0.00	3.125 ± 0.00	2.343 ± 1.11	12.5± 0.00	12.5 ± 0.00
	Fungal sp N241	110	1.56 ± 0.00	23.14 ± 0.00	23.14 ± 0.00	25 ± 0.00	12.5 ± 0.00	1.56 ± 0.00	12.5 ± 0.00
	Unidentified fungal N262	165	>25	>25	>25	>25	>25	>25	>25
	Unidentified fungal N263	94	3.125 ± 0.00	6.25 ± 0.00	3.125 ± 0.00	3.125 ± 0.00	0.78 ± 0.00	6.25 ± 0.00	12.5 ± 0.00
	Unidentified fungal N266	70	3.125 ± 0.00	6.25 ± 0.00	6.25± 0.00	6.25 ± 0.00	3.125 ± 0.00	6.25 ± 0.00	6.25 ± 0.00
	Unidentified fungal N251	110	12.5 ± 0.00	12.5 ± 0.00	25 ± 0.00	6.25 ± 0.00	25 ± 0.00	12.5 ± 0.00	12.5 ± 0.00
	Fungal sp N268	70	ND	23.14 ± 0.00	11.57 ± 0.00	6.25 ± 0.00	25 ± 0.00	1.56 ± 0.00	25 ± 0.00
	Unidentified fungal N276	60	0.78 ± 0.00	3.125 ± 0.00	25 ± 0.00	3.125 ± 0.00	25 ± 0.00	25 ± 0.00	25 ± 0.00
	Unidentified fungal N256	152	6.25 ± 0.00	6.25 ± 0.00	6.25 ± 0.00	3.125 ± 0.00	3.125 ± 0.00	6.25 ± 0.00	12.5 ± 0.00
	Unidentified fungal $N254_1$	52	4.688 ± 0.00	12.5 ± 0.00	12.5 ± 0.00	6.25 ± 0.00	3.125 ± 0.00	6.25 ± 0.00	25 ± 0.00
	Fusarium sp N240	160	1.56 ± 0.00	23.14 ± 0.00	11.57 ± 0.00	6.25 ± 0.00	6.25 ± 0.00	1.56 ± 0.00	12.5 ± 0.00
	Unidentified fungal N441	109	6.25 ± 0.00	6.25 ± 0.00	6.25 ± 0.00	3.125 ± 0.00	3.125 ± 0.00	12.5 ± 0.00	12.5 ± 0.00
	Unidentified fungal N448	70	0.78 ± 0.00	23.14 ± 0.00	5.787 ± 0.00	3.6875 ± 0.00	25 ± 0.00	0.78 ± 0.00	12.5 ± 0.00
	Unidentified fungal N445	200	3.125 ± 0.00	23.14 ± 0.00	11.57 ± 0.00	6.25 ± 0.00	25 ± 0.00	6.25 ± 0.00	25 ± 0.00

	Unidentified fungal N368	123	12.5 ± 0.00	25 ± 0.00	25 ± 0.00	6.25 ± 0.00	25 ± 0.00	12.5 ± 0.00	12.5 ± 0.00
Terminalia catappa	*Corynespora* sp N32	70	1.56 ± 0.00	23.14 ± 0.00	11.57 ± 0.00	6.25 ± 0.00	12.5 ± 0.00	1.56 ± 0.00	12.5 ± 0.00
	Fungal endophyte sp N89	80	1.56 ± 0.00	23.14 ± 0.00	11.57 ± 0.00	6.25 ± 0.00	12.5 ± 0.00	1.56 ± 0.00	12.5 ± 0.00
	Phomopsis sp N114	140	3.125 ± 0.00	23.14 ± 0.00	11.57 ± 00	6.25 ± 0.00	12.5 ± 0.00	1.56 ± 0.00	12.5 ± 0.00
	Guignardia sp N108	110	2.343 ± 1.11	11.57 ± 0.00	5.787 ± 0.00	3.125 ± 0.00	12.5± 0.00	1.56 ± 0.00	12.5 ± 0.00
	Pestalotiopsis sp N75	100	3.125 ± 0.00	5.787 ± 0.00	5.787 ± 0.00	3.125 ± 0.00	25 ± 0.00	1.56 ± 0.00	25 ± 0.00
	Xylaria sp N74	80	4.688 ± 0.00	23.14 ± 0.00	5.787 ± 0.00	6.25 ± 0.00	12.5 ± 0.00	6.25 ± 0.00	6.25 ± 0.00
	Paraconiothyrium variabile $N78_1$	170	1.56 ± 0.00	23.14 ± 0.00	11.57 ± 0.00	6.25 ± 0.00	12.5 ± 0.00	1.56 ± 0.00	25 ± 0.00
	Pestalotiopsis sp N51	70	1.56 ± 0.00	11.57 ± 0.00	5.787 ± 0.00	3.125 ± 0.00	12.5 ± 0.00	1.56 ± 0.00	6.25 ± 0.00
	Unidentified fungal N58	60	3.125 ± 0.00	23.14 ± 0.00	5.787 ± 0.00	6.25 ± 0.00	25 ± 0.00	1.56 ± 0.00	12.5 ± 0.00
	Unidentified fungal N44	70	1.17 ± 0.00	23.14 ± 0.00	11.57 ± 0.00	6.25 ± 0.00	25 ± 0.00	1.56 ± 0.00	25 ± 0.00
	Curvularia sp N101	102	1.56 ± 0.00	4.69 ± 1.80	3.125 ± 0.00	3.125 ± 0.90	1.56 ±0.90	6.25 ± 0.00	6.25 ± 0.00
	Trichoderma sp N97	60	0.78 ± 0.00	23.14 ± 0.00	11.57 ± 0.00	6.25 ± 0.00	25 ± 0.00	1.56 ± 0.00	25 ± 0.00
	Phomopsis sp N81	40	1.56 ± 0.00	11.57 ± 0.00	5.787 ± 0.00	3.125 ± 0.00	25 ± 0.00	25 ± 0.00	25 ± 0.00

	Aspergillus sp N18	103	0.78 ± 0.45	1.56 ± 0.90	3.125 ± 0.00	3.125 ± 0.00	2.343 ± 1.08	6.25 ± 0.00	6.25 ± 0.00
	Aspergillus sp N13	99	1.56 ± 0.00	6.25 ± 0.00	3.125 ± 0.00	3.125 ± 0.00	0.78 ± 0.00	1.17± 0.27	12.5 ± 0.00
	Unidentified fungal N23	75	1.56 ± 0.00	6.25 ± 0.00	6.25 ± 0.00	3.125 ± 0.00	3.125 ± 0.00	12.5 ± 0.00	12.5 ± 0.00
	Unidentified fungal N15	60	1.56 ± 0.00	23.14 ± 0.00	11.57 ± 0.00	6.25 ± 0.00	3.125 ± 0.00	1.56 ± 0.00	12.5 ± 0.00
	Colletotrichum sp N201	10	6.25 ± 0.00	23.14 ± 0.00	11.57 ± 0.00	6.25 ± 0.00	25 ± 0.00	6.25 ± 0.00	25 ± 0.00
	Fungal sp N200	80	3.125 ± 0.00	23.14 ± 0.00	11.57 ± 0.00	6.25 ± 0.00	6.25 ± 0.00	1.56 ± 0.00	6.25 ± 0.00
	Unidentified fungal N191	80	0.39 ± 0.00	5.787 ± 0.00	5.787 ± 0.00	3.125 ± 0.00	12.5 ± 0.00	3.125 ± 0.00	12.5 ± 0.00
	Unidentified fungal N196	58	>25	25 ± 0.00	25 ± 0.00	25 ± 0.00	25 ± 0.00	25 ± 0.00	>25
	Unidentified fungal N221	163	>25	>25	>25	>25	>25	>25	>25
	Unidentified fungal N230$_2$	100	1.56 ± 0.00	12.5 ± 0.00	4.688 ± 2.21	4.688 ± 2.21	3.125 ± 0.00	12.5 ± 0.00	12.5 ± 0.00
Terminalia mantaly	*Diaporthales* sp N229$_1$	119	3.125 ± 0.00	6.25 ± 0.00	3.125 ± 0.00	6.25 ± 0.00	0.78 ± 0.00	6.25 ± 0.00	6.25 ± 0.00
	Diaporthales sp N127	80	1.56 ± 0.00	11.57 ±0.00	5.787 ± 0.00	6.25 ± 0.00	12.5 ± 0.00	1.56 ± 0.00	12.5 ± 0.00
	Phoma sp N116	90	1.56 ± 0.00	23.14 ± 0.00	5.787 ± 0.00	3.125 ± 0.00	6.25 ± 0.00	1.56 ± 0.00	12.5 ± 0.00
	Xylaria laevis N120	57	>25	25 ± 0.00	25 ± 0.00	25 ± 0.00	25 ± 0.00	25 ± 0.00	25 ± 0.00

	Diaporthe sp N162	159	>25	>25	>25	12.5 ± 0.00	>25	>25	>25
	Unidentified fungal $N190_1$	120	0.78 ± 0.00	23.14 ± 0.00	5.787 ± 0.00	3.125 ± 0.00	6.25± 0.00	1.56 ± 0.00	6.25 ± 0.00
	Unidentified fungal N178	134	>25	>25	>25	>25	>25	>25	>25
Reference drugs	Amoxicillin	/	4 ± 00	16 ± 0.00	64 ± 0.00	16 ± 0.00	4 ±0.00	32 ± 0.1.5600	32 ± 0.00
	Ciprofloxacin	/	2 ± 0.00	4 ± 0.00	1 ± 0.00	32 ± 0.00	1 ± 0.00	1 ± 0.00	8 ± 0.00

SA:*Staphylococcus aureus*; EC: *Escherichia coli*; SP: *Streptococcus pneumoniae*; HI: *Haemophilus influenzae*; PA: *Pseudomonas aeruginosa*; KP: *Klebsiella pneumoniae*
